# *Drosophila* lines with mutant and wild type human TDP-43 replacing the endogenous gene reveals phosphorylation and ubiquitination in mutant lines in the absence of viability or lifespan defects

**DOI:** 10.1371/journal.pone.0180828

**Published:** 2017-07-07

**Authors:** Jer-Cherng Chang, David B. Morton

**Affiliations:** Department of Integrative Biosciences, Oregon Health & Science University, Portland, Oregon, United States of America; Children's Hospital of Pittsburgh, University of Pittsburgh Medical Center, UNITED STATES

## Abstract

Mutations in TDP-43 are associated with proteinaceous inclusions in neurons and are believed to be causative in neurodegenerative diseases such as frontotemporal dementia or amyotrophic lateral sclerosis. Here we describe a *Drosophila* system where we have engineered the genome to replace the endogenous TDP-43 orthologue with wild type or mutant human TDP-43(hTDP-43). In contrast to other models, these flies express both mutant and wild type hTDP-43 at similar levels to those of the endogenous gene and importantly, no age-related TDP-43 accumulation observed among all the transgenic fly lines. Immunoprecipitation of TDP-43 showed that flies with hTDP-43 mutations had increased levels of ubiquitination and phosphorylation of the hTDP-43 protein. Furthermore, histologically, flies expressing hTDP-43 M337V showed global, robust neuronal staining for phospho-TDP. All three lines: wild type hTDP-43, -G294A and -M337V were homozygous viable, with no defects in development, life span or behaviors observed. The primary behavioral defect was that flies expressing either hTDP-43 G294A or M337V showed a faster decline with age in negative geotaxis. Together, these observations implied that neurons could handle these TDP-43 mutations by phosphorylation- and ubiquitin-dependent proteasome systems, even in a background without the wild type TDP-43. Our findings suggest that these two specific TDP-43 mutations are not inherently toxic, but may require additional environmental or genetic factors to affect longevity or survival.

## Introduction

Cytoplasmic protein aggregates are a common pathological feature of many neurodegenerative diseases [1]. The TAR DNA binding protein of 43 kDa (TDP-43) has been identified as a major component of the ubiquitinated aggregates in neurons of patients with either frontotemporal lobar degeneration (FTLD) or amyotrophic lateral sclerosis (ALS) [2, 3]. The subsequent identification of mutations in TDP-43 in FTLD and ALS patients also identified this gene as a causative agent in these diseases [4]. In addition, TDP-43 positive inclusions have also been reported as important characteristics in other neurodegenerative diseases, such as Alzheimer’s disease, Parkinson’s disease and Huntington disease [5]. All these findings appear to directly link the presence of TDP-43 inclusions to the etiology of a variety of neurodegenerative diseases [6].

TDP-43 is a member of the heterogeneous nuclear ribonucleoprotein family [7], is expressed ubiquitously in neurons, glia and other cell types and plays a significant role in many aspects of RNA metabolism, such as RNA alternative splicing, stability, transcriptional regulation, mRNA transport and translation [8]. This protein is strongly conserved in both vertebrates and invertebrates suggesting an essential role of TDP-43 in cell physiology and several model systems have demonstrated lethality caused by loss of function of TDP-43 [9–15]. Similarly, several studies have used cellular, invertebrate and vertebrate animal models, to either over-express or conditionally express wild type or mutant TDP-43 to reproduce the TDP-43 aggregates similar to those found in ALS and FTLD (reviewed in [16–21]. These animal models strongly implicate a pathogenic role of TDP-43 in neurodegenerative diseases [16, 22–24]. Although these studies have revealed many aspects of TDP-43 biology, there are a number of important issues that remain to be resolved concerning the relationship between the molecular phenotypes and disease progression. In most cases involving mutant TDP-43, the associated disease onset occurs in middle age or older. This contrasts to rapid disease onset observed in models involving overexpression of TDP-43. Additionally, there are several reports of familial TDP-43 mutations where there appears to be a lack of concordance between the presence of the mutation and disease [25–31]. Furthermore, different families with the same TDP-43 mutations or patients within the same family have significant differences in age of disease onsets [27, 32, 33]. These clinical observations not only suggest that TDP-43 proteinopathies are polymorphic, but also suggests that TDP-43 mutations might not the sole factor for the disease onset and lethality.

Using CRISPR/Cas9 genome editing techniques, we generated three fly lines where the coding region of the endogenous TDP-43 orthologue (TBPH) was replaced with either wild type human TDP-43 or one of two mutations; hTDP-43 G294A (identified as a sporadic mutation) and hTDP-43 M337V (a familial mutation). In contrast to other studies, in which mutated TDP-43 is expressed in addition to the endogenous TDP-43, these three fly lines express wild type or mutant hTDP-43 in the absence of expression of the fly *tbph* gene. No significant phenotypes were observed in flies expressing wild type hTDP-43 compared to control flies, suggesting hTDP-43 can fully replace the functions of the *Drosophila* gene. Also in contrast to TDP-43 over-expression models, the fly lines carrying ALS-associated mutations were viable and only exhibited mild behavioral phenotypes. These results suggest that these two TDP-43 mutations, G294A and M337V, are not inherently toxic, but might require additional genetic and/or environmental factors to exhibit disease-like phenotypes.

## Materials and methods

### Fly stocks

All *Drosophila* stocks were reared at 25°C using standard procedures [34]. The following fly strains were obtained from the Bloomington stock center (http://flystocks.bio.indiana.edu/): y[1] M{vas-Cas9.RFP-}ZH-2A w[1118] was the parental line used for CRISPR/Cas9 injections and y[1] w[67c23] P{y[+mDint2] = Crey}1b; sna[Sco]/CyO carrying the Cre recombinase was used to remove the DsRed cassette in the transgenic flies. The hTDP-43 transgenic flies and the parental flies were crossed with *w*^*1118*^ at least five times for minimizing the nonspecific effects caused by CRISPR/Cas9 [35] and to reduce differences in the genetic background between lines.

### Generation of the hTDP-43 replacement fly lines

**Fig 1:**
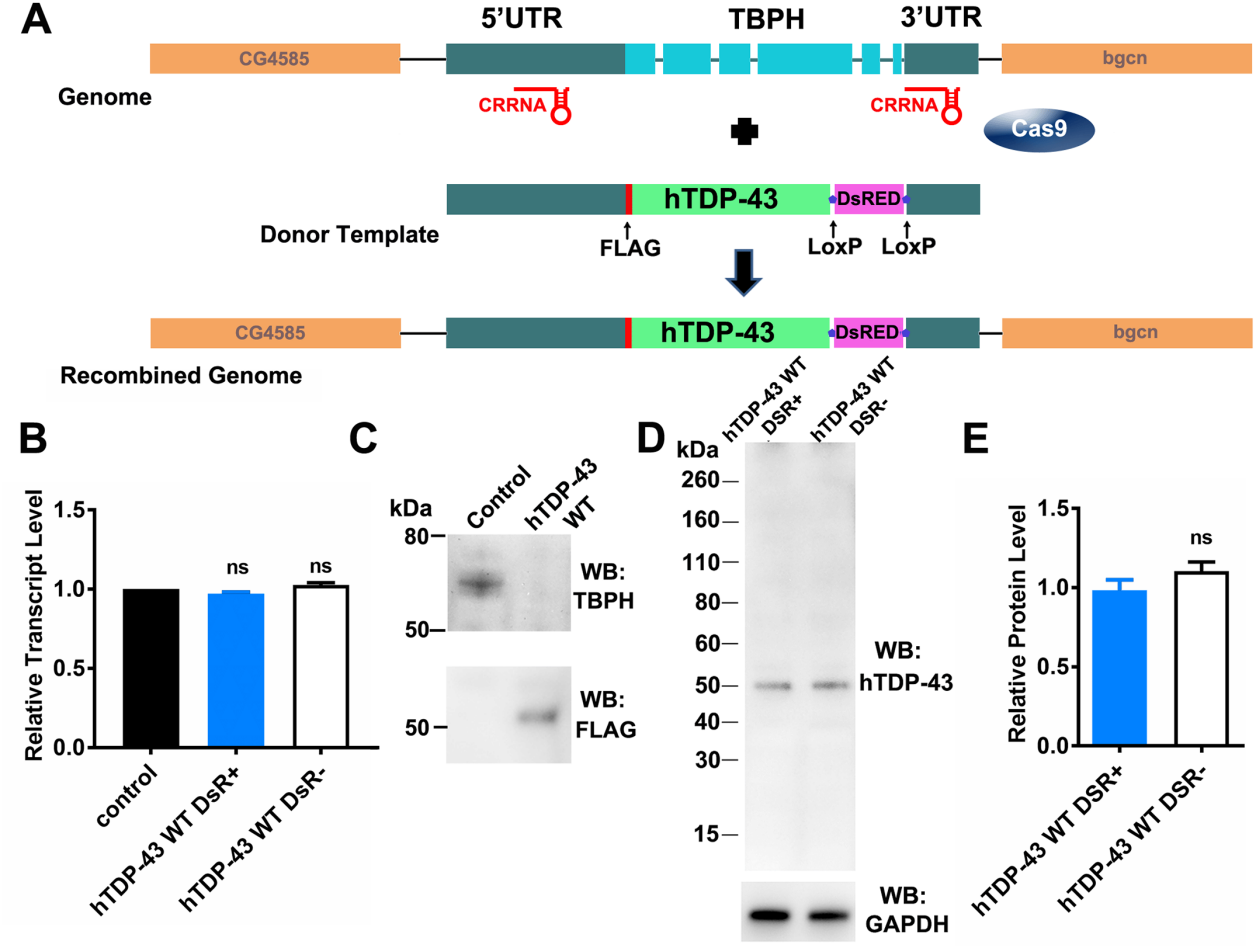
Replacement of the *Drosophila* TBPH gene with human TDP-43 (hTDP-43) using the CRISPR/Cas9 genome editing system. **A**. Schematic diagram showing the relative positions of chimeric RNA (CRRNA) hybridization site in the TBPH gene and the donor template containing the 5' UTR of TBPH, hTDP-43 cDNA, the DsRed cassette flanked by LoxP sites and the 3'UTR of TBPH. **B**. Real time RT-PCR using a probe against the 5'UTR of TBPH showing unchanged levels of transcript in control flies, and flies expressing wild type hTDP-43 either retaining the DsRED cassette (hTDP-43 DsR+) or with it excised (hTDP-43 DsR-). Mean and standard error from six independent samples are shown. One way ANOVA showed no significant (ns) differences between the genotypes. **C**. Representative immuno-blot from adult heads showing TBPH present in control flies but not transgenic hTDP-43 WT flies (upper panel) and hTDP-43 (detected with an anti-FLAG antibody) present in flies expressing wild type hTDP-43 but not in control flies (lower panel). **D**. Representative hTDP-43 immuno-blot from adult heads showing a single band of the expected molecular weight, with similar protein levels in flies with (DSR+) or without (DSR-) the DsRED cassette. The lower panel shows the levels of GAPDH used as a loading control. **E**. Quantification of immunoblots. The intensity of the hTDP-43 immuno-reactive band was quantified, normalized to the intensity of GAPDH band and compared to the ratio obtained for the DsRED positive fly. T-test showed no significant (ns) change when the DsRed cassette was excised.

A schematic diagram of the strategy used to generate flies in which the coding region of TBPH was replaced with a cDNA for human TDP-43 (hTDP-43) is shown in Fig 1. Fly embryos expressing the nuclease Cas9 under the control of the vasa promoter (vas-Cas9; y[1] M{vas-Cas9.RFP-}ZH-2A w[1118]/FM7a, P{w[+mC] = Tb[1]}FM7-A) were injected with two plasmids by BestGene (Chino Hills, CA, USA). One plasmid contained a donor template coding for the hTDP-43 cDNA sequence and a red fluorescent protein under the control of an eye-specific promoter flanked by portions of the TBPH 3’ and 5’ UTR was injected at a concentration of 500 ng/μl, and the other contained two guide RNAs and injected at a concentration of 200 ng/μl. The guide RNAs target cas9 to sites on either side of the TBPD coding region, resulting in two double strand breaks which are repaired by homologous recombination using the donor template.

To generate the donor template, the fly codon-optimized human TDP-43 cDNA sequence was synthesized (GeneArt, Lifetechnologies; CA, USA) and cloned into pCMV-3FLAG to produce the FLAG-hTDP-43 DNA fusion. This FLAG-hTDP-43 fragment was ligated to the 3’ end of *Drosophila TBPH* 5’UTR (amplified by primers 5’ Homology Arm-S and -R, Table 1), and sub-cloned into the AarI sites of 5’ MCS in pHD-DsRED attp vector [36]. The 3’ UTR of *TBPH* (amplified by primers 3’ Homology Arm-S and -R, Table 1) was incorporated through the SapI sites of 3’ MCS in pHD-DsRED attp vector. To incorporate ALS/FTD associated TDP-43 mutations into the donor template, primers against hTDP-43 G294A or M337V were designed through the NEBaseChanger web tool. The pHD-DsRED TDP-43 mutant forms were generated, followed by the instruction of Q5 site-directed mutagenesis kit (New England Biolabs; MA, USA).

**Table 1:** Primers used in this study.

CRRNA-5UTR	CTTCGTTTTAGTATATCTACCCGC
CRRNA-3UTR	CTTCGAACCACATCATTGGGTGAC
5’ Homology Arm S	AAAACACCTGCAAAATCGCGGAGCCCACCATGATGATACC
5’ Homology Arm R	CCCAAGCTTTTTCCTAGAAGGCTGCTAGAAACGAG
5’ Homology Arm S	AAAAGCTCTTCATATACCCAATGATGTGGTTAATGTGGTT
5’ Homology Arm R	AAAAGCTCTTCAGACCGCCGTCCCGCCAA
GT-4585	TGCGGTCGCTCCCCAAAGTGTCAAAAAT
GT-bgcn	CCTGGCGAATACTCCGGTTTGGTG
qRT-TBPH S	GTCAGGCGATACATACAAAGGAAAAGAA
qRT-TBPH R	CGGTGCGAAATAGGAATCAGGTAA
qRT-EF2 S	GCGTTCACCCTCAAGCAGTTCT
qRT-EF2 R	AGCGTTTGTTGTCAGCCTCTTTCT
qRT-9165 S	GCCGTGCTGGAGCGAGAAGAT
qRT-9165 R	CTGGGCTGTGCGACGAAGAGAT
qRT-SDha S	TCTTCGCCGGTGTTGATGTGAC
qRT-SDha R	GTGATTACCTGGCCGCGATAGTTAGT
qRT-CALR S	AAGGAGAATTTCGACAACGGTAAGTGGTAG
qRT-CALR R	GGGAACAAGCGGGGGAGTGC

The guide RNA sequences were designed using the *Drosophila* RNAi Screening Center CRISPR2 web tool (http://www.flyrnai.org/crispr2/). The sequences selected were located in the intron within the TBPH 5’ UTR and the 3’ end sequence was adjacent to the stop codon of the TBPH coding region. These two crRNA (see Table 1, CRRNA-5UTR and CRRNA-3UTR) were cloned into two sibling vectors, pBFv-U6.2 and pBFv-U6.2B, respectively [37]. Finally, these two independent crRNA cassettes were fused into a single plasmid for increased injection efficiency [37].

The survival rates for the injected embryos were 50%, 45% and 45% for wild type hTDP-43, hTDP-43 G294A and hTDP-43 M337V, respectively, which gave final transformation efficiencies(based on the presence of DsRed signal) of 0.5%, 3% and 1%.

### Genotyping of the hTDP-43 replacement flies

The sequence spanning the 5’ upstream gene, CG4585 to the downstream gene *bgcn*, was amplified by Q5 high-fidelity polymerase (New England Biolabs) using primers GT-4585 and GT-bgcn (see Table 1) and cloned into the TA-vector for sequencing, to confirm the expected sequence of the TBPH genomic region. Original TBPH genomic length: 7527bp; hTDP-43 DsRed positive (DsR+): ~8500bp; hTDP-43 DsRed (DsR-): ~7200bp.

### Generation of DsRED minus flies

To generate the fly lines lacking the eye-specific DsRed cassette, fly lines expressing wild type hTDP-43 were crossed with flies expressing Cre recombinase. The DsRED cassette within the donor template is flanked by two loxP sites, and can be excised in the presence of Cre recombinase. Offspring from this cross were screened for lack of red fluorescent eyes, crossed to a second chromosome balancer line and the resulting progeny selected for loss of the balancer chromosome and then genotyped to confirm the presence of hTDP-43 and absence of TBPH.

### Real time reverse transcription (RT)-PCR

Total RNA was extracted using Trizol reagent (Life Technologies, CA, USA), pre-cleaned by DNaseI and cDNA synthesis performed using SuperScript III (Life Technologies, CA, USA). To determine the relative expression of TBPH and hTDP-43 in the control flies and flies expressing wild-type hTDP-43, hTDP-43 G294A and hTDP-43 M337V, primer pairs against the 5’ UTR of *TBPH* gene, and also three internal controls, EF2b, CG9165 and SDha were designed using Primer3 (See Table 1). PCR reactions were performed in a StepOne thermocycler (Life Technologies) through the use of Power SYBR^®^ Green PCR Master Mix (Applied Biosystems, Warrington, UK). The PCR reaction conditions were: 95°C for 10 min, followed by 40 cycles of 15 sec at 95°C, 30 sec at 63°C and 40 sec at 72°C.

The mean value of TBPH Ct minus the mean set of controls was the delta Ct (ΔCt) values. Three replicates of ΔCt of individual loading control to all the samples were calculated for the means and were normalized to the loading control, EF2. Finally, the normalized mean values were normalized to the values of control for relative expression levels.

### Immunoblotting

Antisera used for immunoblotting are listed as below: mouse anti-FLAG antibodies (1:5000; Sigma, Saint Louis, MO, USA), mouse anti hTDP-43 2E2-D3(1: 2000; Abnova, Taipei, Taiwan), mouse anti phospho(403/404)-TDP-43(1:3000; Proteintech, Rosemont, IL) rabbit anti K48-linkage ubiquitination (1:1000; Abcam, Cambridge, MA), mouse anti GAPDH (1: 1000; Santa Cruz, Dallas, Texas), goat anti mouse peroxidase conjugate (1:10000; Jackson Immunoresearch, West Grove, PA, USA), goat anti mouse (1:10000; Jackson Immunoresearch) and bovine anti goat peroxidase conjugate (1:10000; Jackson Immunoresearch).

Adult fly heads were dissected on an ice-cold plate, washed with PBS followed by 70% ethanol and homogenized in lysis buffer (50 mM Tris-HCl pH 7.5, 150 mM NaCl, 1% TritonX100, 1mM EDTA, 5% glycerol, 2 mM AEBSF, 0.3 μM aprotinin, 130 μM bestatin, 14 μM E-64, and 1 μM leupeptin) and centrifuged at 1,000 × g for 5 min at 4°C. The supernatant was kept as the Tris-soluble fraction, and the pellet was extracted with 0.5% SDS, followed by 10,000 × g for 5 min at RT. The supernatant was kept as SDS soluble fraction. These Tris-soluble and SDS-soluble fractions were denatured in 1X Laemmli sample buffer at 85°C and proteins fractionated by SDS-PAGE. Following transfer to polyvinylidine difluoride membranes (Pall life science, NY, USA), the membranes were blocked with either 0.1% gelatin in PBST (PBS containing 0.05% tween-20) or 3% non-fat milk and incubated with either anti-FLAG or anti-hTDP-43 antisera followed by HRP-conjugated goat-anti mouse antisera. The proteins were then detected with enhanced chemiluminescence DuoLuX (Vector laboratories, Inc., Burlingame, CA, USA). The same blot was stripped with stripping buffer (Thermo, Rockford, USA) at room temperature for 10 mins, followed by blocking, probed with 1:1,000 anti-GAPDH antisera, incubation of 1:10,000 HRP-conjugated goat-anti rabbit antisera (Jackson Immunoresearch) and the detection with enhanced chemiluminescence (Invitrogen). The relative TDP-43 levels were normalized to GAPDH levels by use of Gel plugin of ImageJ.

### Immunofluorescence

To visualize the distribution of FLAG-hTDP-43 in the central nervous system (CNS), 7 days-old flies were prefixed in 4% formaldehyde in 1X PBS, 1%TritonX-100 for 20 min at room temp. Dissection of the CNS was performed in ice-cold PBS and the tissue post-fixed in 4% formaldehyde in 1X PBS, 0.3%TritonX-100 (PBSTX) for 20 min. The dissected tissues were then blocked with 5% normal goat serum (NGS) in PBSTX overnight, followed by incubation in mouse anti-FLAG antibody (sigma Aldrich; 1:500) overnight at 4°C in PBSTX. After washing 5X with PBSTX, the tissue was incubated in 1:500 AlexaFluor 488 goat anti mouse IgG (Jackson Immunoresearch) overnight at 4°C, and then washe 5Xwith PBSTX. The tissue was then rinsed in a series of concentrated glycerol and mounted in 50%Vectashield (Vector Laboratories, INC., Burlingame, CA). Images were acquired with an Olympus Fluoview FV1000 confocal microscope using the same setting of Olympus Fluoviewer3.1 for experimental and control samples. For the cytoplasmic FLAG-hTDP-43 signal, the fluorescence of the pixels outside the nucleus (delineated by DAPI staining) were quantified by use of the histogram plugin of FIJI, following subtraction of the background signal, and then normalized to the total pixels within the cells.

For FLAG/FMR1 double staining on adult CNS, procedures were the same as described above except the primary antibodies were changed to: rabbit anti-FLAG (sigma Aldrich; 1:500) and mouse anti FMR1 (Developmental Studies Hybridoma Bank; 1:100) and the secondary antibodies: 1:500 AlexaFluor 488 goat anti rabbit IgG (Jackson Immunoresearch) and 1:500 AlexaFluor 647 goat anti mouse IgG (Jackson Immunoresearch). For phospho(S409/S410)TDP/Ub-K48 double staining primary antibodies: rat anti-phospho(S409/S410)TDP antibody (Millipore; 1:250) and rabbit anti ubiquitin K-48 (Abcam, 1:100); secondary antibodies: 1:500 AlexaFluor 488 donkey anti rabbit IgG (Jackson Immunoresearch) and 1:500 AlexaFluor 647 goat anti rabbit IgG (Jackson Immunoresearch).

### Phenotyping

All the hTDP-43 replacement flies were outcrossed to the control flies (described above) at least five times to minimize the effects of differing genetic backgrounds and potential CRISPR off-target effects. To determine if there were any differences in developmental time mated females from each line were allowed to lay eggs for 24 hrs and the time taken for the progeny to eclose was recorded. The longevity of each line was assessed by collecting virgin males and females of each line and placing them in separate vials of 10–15 animals in each vial. Vials were inspected for dead flies every other day and the remaining flies placed in new vials each week. Fecundity was assessed by placing a single virgin male and female in a vial for 7 days and then counting the total number of adult flies produced from each vial.

### Behavioral assays

Larval locomotion was determined for each line as previously described [11] and adult activity assessed in an activity recorder as previously described [38].

Negative geotaxis assays were performed by placing flies (ranged from 1~28 days-old of age) in separate vials (10 flies per vial), one day before the assays. Flies were transferred to adapted 25 ml tubes to be used as vertical climbing columns. Flies were placed in a 25°C incubator for recovery for 30 min before the experiment and then tapped to the bottom of the tubes and allowed to climb for 60 s and a digital image captured of the entire tube. The whole climbing tubes was captured as an image. The number of flies in the top one tenth (n_top_) and the bottom one tenth (n_bottom_) of the tubes was recorded. A performance index (PI) was calculated as follows: ½[(n_total_ + n_top_ − n_bottom_)/n_total_] as described previously [39]. The mean PI of each genotype was calculated from at least three groups of flies.

## Results

### Generation of novel fly lines that express hTDP-43 at equivalent levels to the endogenous TDP-43 orthologue

Previous studies using *Drosophila* to model TDP-43 proteinopathies have shown that loss of function of TBPH, the *Drosophila* TDP-43 orthologue, is lethal [9–11, 15]. Similarly, over-expression of hTDP-43 in fly models also highlights the toxicity of over-expression of both wild type and mutant hTDP-43 [40–45]. To minimize the effects of over-expression, we have developed an alternative method of expressing hTDP-43 cDNA under the control of the endogenous TBPH regulatory regions in the absence of endogenous TBPH expression, using the CRISPR/Cas9 genome editing technique (Fig 1A). Immunoblots from the resulting fly line demonstrated the absence of TBPH and presence of FLAG-hTDP-43 (Fig 1C). The donor template used to replace the TBPH coding region with hTDP-43 also included a sequence for expressing a red fluorescent protein (DsRed) under the control of an eye-specific promoter to identify flies that incorporated hTDP-43 (Fig 1A). To verify that the presence of the DsRed cassette had no effect on the expression of hTDP-43, we also generated a DsRed free-hTDP fly line by excising the DsRed cassette utilizing the flanking loxP sites (Fig 1A). We assessed the levels of hTDP-43 and TBPH transcripts by quantitative RT-PCR using a probe against the 5’UTR of TBPH, which is common to all three of these lines. These results showed that there were no differences in expression between flies expressing TBPH (control) and wild type hTDP-43 with (hTDP-43 WT DSR+) or without (hTDP-43 WT DSR-) the DsRed cassette (Fig 1B). Similarly, the protein levels of hTDP-43 showed no difference in flies that retained or had the DsRed cassette excised (Fig 1D & 1E), suggesting that the DsRed cassette did not interfere with the expression of hTDP-43.

### Reciprocal changes in TDP-43 transcript and protein levels in flies expressing mutant hTDP-43

Using the same strategy, we generated two additional fly lines carrying disease causing mutations in hTDP-43 along with the DsRed marker. One of these, hTDP-43 G294A, was identified in a sporadic ALS case, with disease onset at 65 years of age [32, 46]. The other, hTDP-43 M337V, was identified in three cases of familial ALS, with disease onset ranging from 37 to 55 years of age [32, 33, 47].

**Fig 2:**
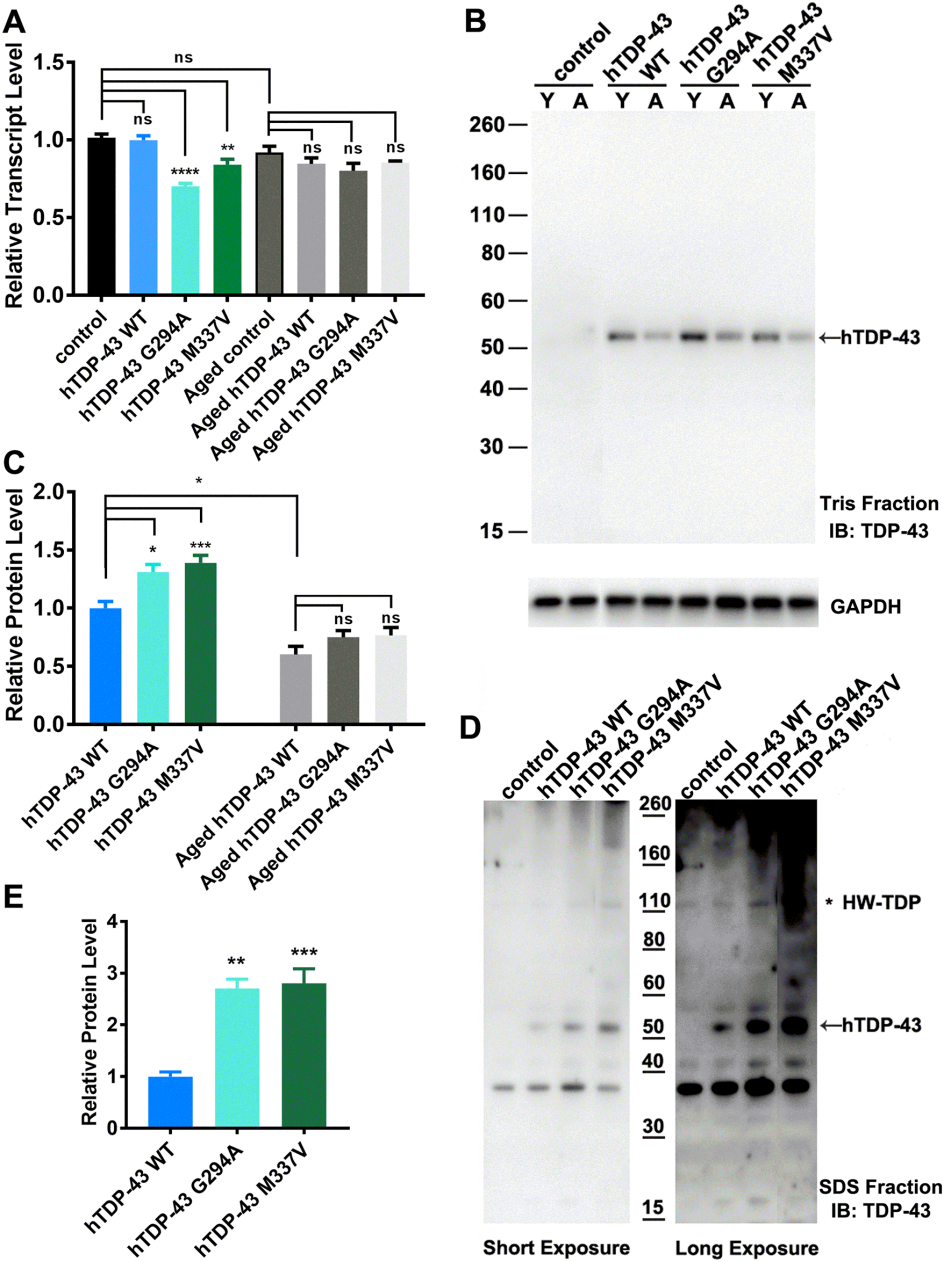
TDP-43 mutations result in changes of transcript and protein level in young flies, but not in aged flies. **A**. Transcript levels. Real time RT-PCR was carried out on the total RNA from adult heads of young (7 day old) and aged (28 day old) animals. For each sample, primers to the 5’ UTR of TBPH and three separate housekeeping genes were used (EF2, CG9165 and SDha) and the relative expression levels averaged across each housekeeping gene and then normalized to the relative expression in control flies. No significant difference between control flies and flies expressing wild type hTDP-43 was seen; whereas flies expressing either mutant hTDP-43 had significantly lower levels of hTDP-43 transcript. However, these transcript variations between hTDP-43 wild type and mutant flies were diminished in aged samples. Mean and SEM from three independent samples are shown, ** p<0.01, **** p<0.0001(ANOVA). **B-E**. Protein levels. Adult fly heads from each genotype were homogenized in Tris buffer (see Methods), centrifuged and the supernatant (Tris fraction) analyzed by immunoblot with an anti-hTDP-43 antiserum and anti-GAPDH as a loading control. The pellet was re-homogenized in SDS buffer, centrifuged and the supernatant (SDS fraction) analyzed by immunoblot in parallel. **B**. Representative immuno-blot for Tris-soluble fractions of young (Y) and aged (A) flies for each genotype. **C**. Quantification of Tris-soluble fraction showing increased levels of hTDP-43 in both lines expressing mutant hTDP-43 relative to flies expressing wild type hTDP-43 (WT hTDP-43). The intensity of the 50kDa band was digitized and the relative intensity compared to that of GAPDH calculated. For each blot the relative expression was normalized to the expression of hTDP-43 in flies expressing wild type hTDP-43. The levels of hTDP-43 in flies expressing mutant hTDP-43 was significantly elevated relative to flies expressing wild type hTDP-43. Notably, the hTDP-43 protein level was decreased with age in all fly lines. There was no difference in hTDP-43 levels among all the aged genotypes. *p<0.05, ** p<0.01, (ANOVA) (mean and SEM, n = 3–9). **D**. Representative blot for the SDS-soluble fraction for each genotype. The two panels represent different exposures with the longer exposure revealing increased levels of high molecular weight species of hTDP-43 in the flies expressing mutant hTDP-43. **E**. Quantification of immunoblots for the SDS-soluble fractions showing increased levels of hTDP-43 in both lines expressing mutant hTDP-43 relative to flies expressing wild type hTDP-43 (WT hTDP-43). The intensity of the ~50 kDa band was digitized and the relative intensity compared to that of GAPDH calculated. For each blot the relative expression was normalized to the expression of hTDP-43 in flies expressing wild type hTDP-43. The levels of hTDP-43 in flies expressing mutant hTDP-43 was significantly elevated relative to flies expressing wild type hTDP-43. ** p<0.01, (ANOVA) (mean and SEM, n = 3).

To determine the expression level of hTDP-43 we used real-time RT-PCR with a probe against the 5’UTR of TBPH, which is preserved in all four lines and tested young (~ 7 days post eclosion) and aged (~ 28 days post eclosion) flies. For both young and aged flies, these results clearly show that there was no difference in the levels of TBPH/hTDP-43 transcript between control flies (expressing TBPH) and flies expressing wild type hTDP-43. By contrast, both mutant lines had slightly reduced transcript levels in young flies (Fig 2A), however, these transcriptional differences were absent in aged flies. In addition, there was no significant difference in the transcript levels between young and aged control or hTDP-43 WT flies.

To assess the levels of hTDP-43 protein expressed in these lines we separated the total CNS proteins of all fly lines into two fractions, Tris-soluble and SDS-soluble fractions, and then analyzed these fractions on immuno-blots using an antiserum against hTDP-43. The Tris-soluble fraction revealed a single immunoreactive band at the expected size for hTDP-43 in the three lines expressing hTDP-43 (Fig 2B). No immunoreactive band was detected in control flies due to the lack of cross reactivity of the antiserum with TBPH. In contrast to the results observed from the real time RT-PCR, both lines expressing mutant hTDP-43 showed higher levels of hTDP-43 protein compared to the flies expressing wild type hTDP-43 in this fraction in young animals (Fig 2B & 2C). The SDS-soluble fraction from young animals also showed elevated levels of hTDP-43 in the two mutant lines (Fig 2D & 2E) and in addition revealed the presence of increased levels of high molecular weight species of hTDP-43 in the two mutant hTDP-43 expressing lines (Fig 2D). Interestingly, aged flies expressing either wild type or mutant hTDP-43 showed decreased hTDP-43 protein levels as the flies aged in both the Tris (Fig 2B & 2C) and SDS (S1 Fig) fractions.

### Ubiquitination and phosphorylation of mutant hTDP-43

**Fig 3:**
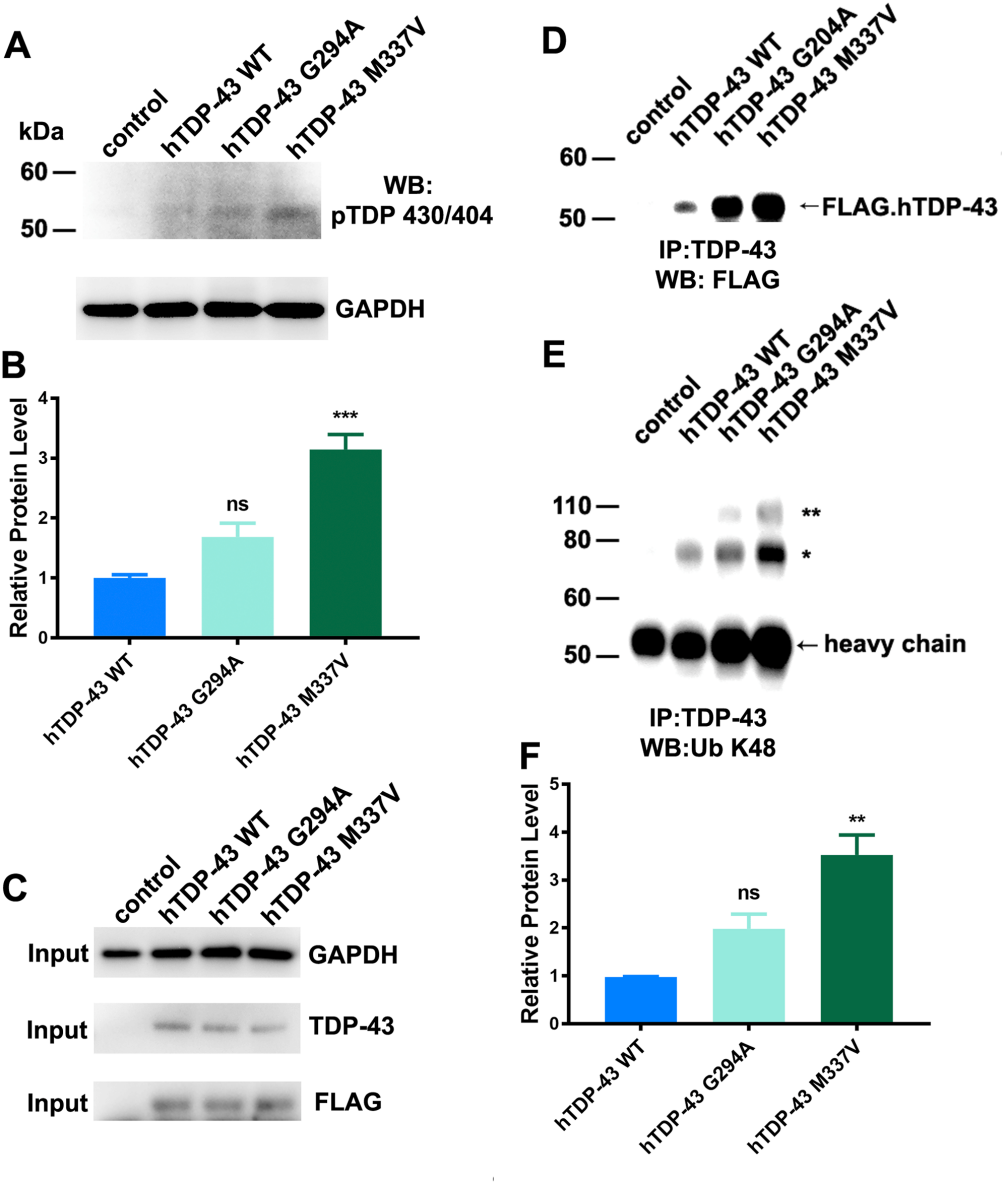
Characterization of the phosphorylated and ubiquitinated hTDP-43 in flies expressing mutant and wild-type hTDP-43. **A**. Representative immunoblots of head lysates blotted with anti-pTDP and anti-GAPDH. **B**. Quantification of the immunoreactive pTDP signal, normalized to the signal of GAPDH. **C**. Input levels. Representative immunoblots of input samples probed with anti-GAPDH, -TDP-43 and -FLAG antiserum. **D-F**. Immunoprecipitated samples. Adult fly heads from each genotype were homogenized in Tris buffer (see Methods), centrifuged and the supernatants immunoprecipitated with anti-TDP-43 and protein G agarose and analyzed by immuno-blots. **D**. Representative immuno-blot probed for FLAG.hTDP-43. Both mutant fly lines (G294A and M337V) revealed the full length hTDP-43 (~50 kDa), and also a truncated fragment (~40 kDa). Samples from both mutants also revealed significantly higher levels of hTDP-43 compared to flies expressing wild type hTDP-43. **E**. Representative immuno-blot probed for the K48 specific ubiquitin linkage (Ub K48). An approximately 80-kDa UB K48 signal was present in all of the transgenic hTDP-43 fly samples (*), whereas a larger, approximately 110-kDa Ub K48 band was only detectible in samples from the mutant hTDP-43 lines. **F**. Quantification of the 80-kDa Ub K48 signal shows that the sample of the M337V mutant has the significant increase of Ub K48 intensity. ** p<0.01,***p<0.001 (ANOVA) (mean and SEM, n = 3).

To characterize the molecular phenotypes further, we used antiserum specific to phosphorylated TDP (pTDP) and examined immunoblots of head lysates of the fly lines. In addition, we used an ubiquitination linkage-specific K48 antibody to examine immunoprecipitated protein, pulled-down by a TDP-43 antibody. When the lysates were blotted with the pTDP-43 antibody, a band of~53 kDa was visible in all three of the transgenic fly lines and notable was increased in flies expressing hTDP-43 M337V (Fig 3A and 3B). When the immunoprecipitated samples were immuno-blotted with anti-FLAG antiserum, immuno-reactive bands of ~50-kDa were detected in all three hTDP-43 transgenic fly samples, and the levels of protein in the mutant samples were highly enriched (Fig 3D). We also blotted these samples with antisera against the K48-specific ubiquitin linkage (Ub-K48). Two major immuno-reactive bands were detected, one at ~80-kDa, which was present in all three of the hTDP-43 transgenic fly lines; while the other, at ~110-kDa, was present only in mutant samples (Fig 3E). It was notable that the signal for both Ub-K48 bands was always the strongest for the hTDP-43 M337V samples (Fig 3F).

### Increased cytoplasmic puncta in neurons from flies expressing mutant hTDP-43

**Fig 4:**
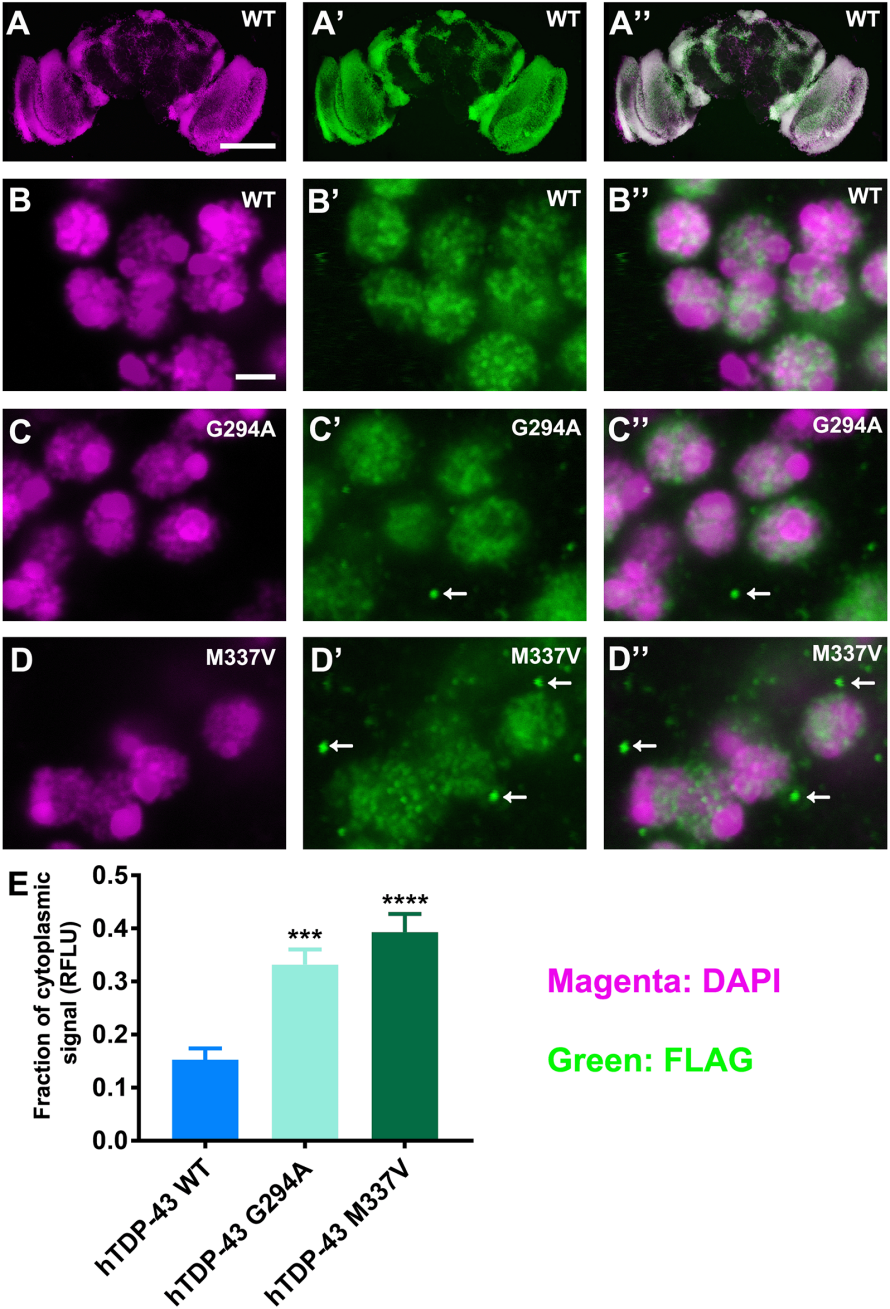
Increased cytoplasmic localization of hTDP-43 in flies expressing mutant hTDP-43. **A**. Widespread expression of hTDP-43 in adult brain. Adult brains were fixed and stained with an anti-FLAG (FLAG) antiserum to label FLAG-hTDP-43 (A’) and DAPI to label nuclei (A). Merged images are shown in the third column (A”). The boxed area indicates the region shown at higher magnification in B-D. **B-D**. Higher magnification images from flies expressing wild type hTDP-43 (**B**), hTDP-43 G294A (**C**) and hTDP-43 M337V (**D**). For both mutant lines there is an increase in FLAG-positive puncta observed in areas that are DAPI negative (arrows in C’ and D’). **E**. Quantification of the non-nuclear hTDP-43 in adult brain. Cytoplasmic FLAG fluorescence was normalized to the total FLAG fluorescence, and over 100 neurons were quantified for individual prep. As the result the cytoplasmic FLAG is significantly elevated in flies expressing hTDP-43 G294A and M337V. (ANOVA, *** p<0.0005,****p<0.0001, n = 8). Scale bar is 50 μm in A and 2μm in B.

We also examined the expression of hTDP-43 in neurons in the adult CNS using immuno-fluorescence to localize the FLAG-tag, fused at the N-terminus of hTDP-43 (Fig 4). These results showed that most, if not all, neurons expressed hTDP-43, similar to results reported for TBPH expression in the adult CNS [10, 48]. In flies expressing wild type hTDP-43, the majority of the FLAG-TDP-43 signal was located in the nucleus (Fig 4B). By contrast, in flies expressing mutant hTDP-43, although the majority of the signal was detected in nuclei, we also observed many neurons that contained small fluorescent puncta located in the cytoplasm (Fig 4C & 4D). To quantify this, we determined the fraction of cytoplasmic versus total FLAG immuno-fluorescence. As expected, in flies that expressed wild type hTDP-43 there was a low level (about 10%) of FLAG-immuno-fluorescence localized in the cytoplasm, as judged by the lack of overlap with the DAPI signal. This is consistent with previous studies showing that most of the TDP-43 is located in the nucleus with some protein shuttling between the nucleus and the cytoplasm [49]. By contrast, in flies expressing mutant hTDP-43 G294A or its M337V mutant there were significant increase in the amount of TDP-43 localized to the cytoplasm with approximately 30% in the cytoplasm (Fig 4E). The majority of this cytoplasmic fluorescent signal was present as small (approximately 0.2μm) puncta in the two mutant lines (Fig 4C & 4D arrows).

The punctate nature of the cytoplasmic TDP-43 in the flies expressing mutant hTDP-43 was reminiscent of stress granules. To determine whether the observed cytoplasmic puncta shared characteristics of stress granules, we co-labeled the CNS using anti-FLAG antiserum as well as an antiserum against *Drosophila* fragile X mental retardation 1 (FMR1), a component of stress granules in *Drosophila* [50–52]. Although the majority of the FMR1 staining was present as cytoplasmic puncta as expected (Figure A in S2 Fig), none of these puncta co-localized with the FLAG puncta in neurons in flies expressing hTDP-43 M337V (Figure B in S2 Fig), suggesting the TDP-43 cytoplasmic puncta are either not stress granules or are RNA granules that do not contain FMR1.

### Enrichment of phosphorylated hTDP-43 and ubiquitin puncta within neurons in flies expressing mutant hTDP-43

**Fig 5:**
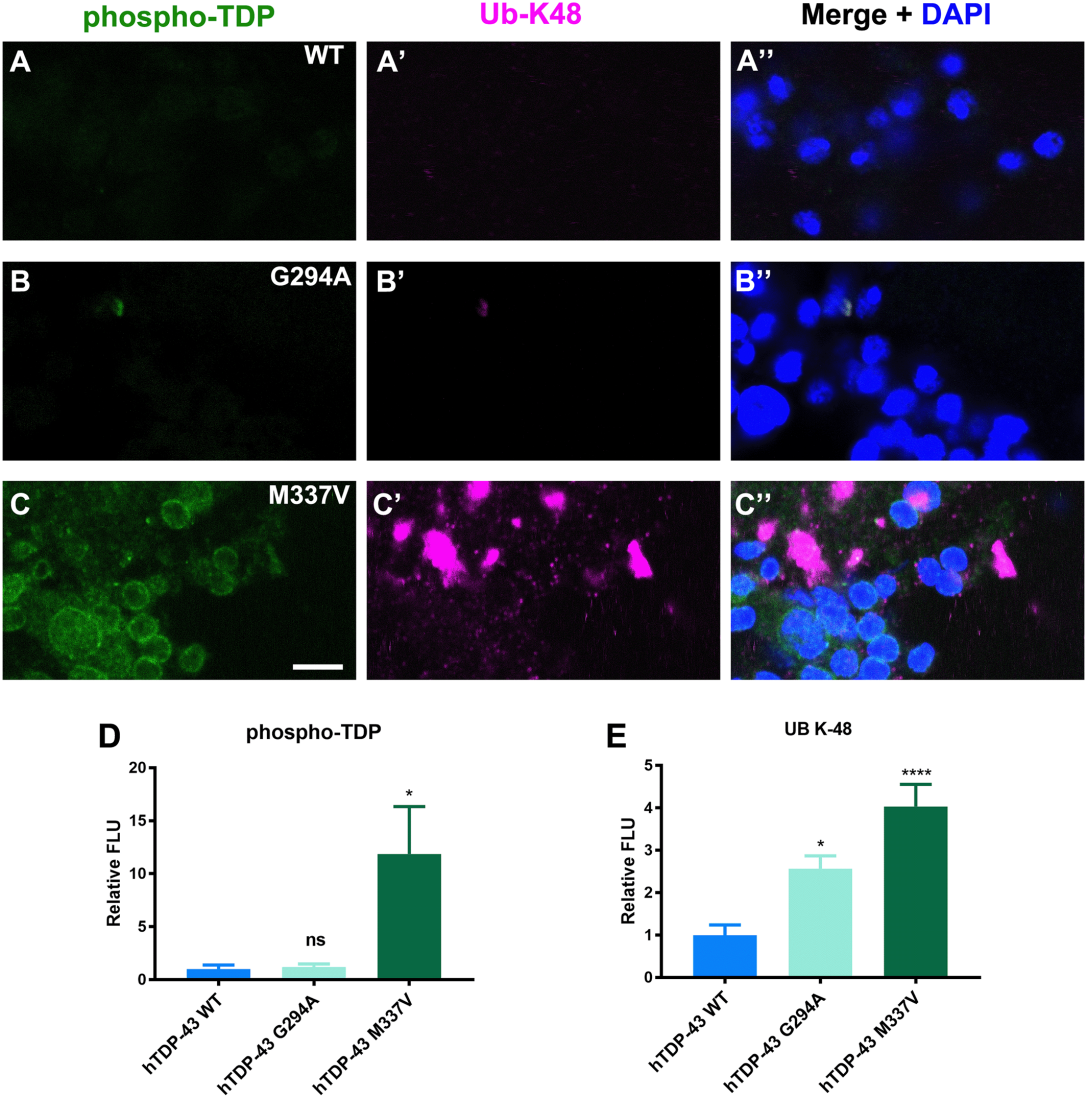
Global phosphorylation of hTDP-43 and increased ubiquitin puncta in neurons in flies expressing hTDP-43 M337V. Adult brains of flies expressing wild type (A) and mutant hTDP-43 (B&C) were fixed and double stained with anti-phospho (409/410)TDP (green) and an anti-ubiquitin linkage K48 (magenta) antiserum to label ubiquitin (A’, B’ and C’) and DAPI to label nuclei (blue). Merged images are shown in the third column. **A**. No significant signal was detected in either phospho-TDP or the Ub-K48 channels in flies expressing wild type hTDP-43. **B**. Flies expressing hTDP-43 G294A showed no significant signal in either phospho-TDP or the Ub-K48 channels. Occasionally, cytoplasmic puncta was double labeled with phospho-TDP and Ub-K48. **C**. Representative images from flies expressing hTDP-43 M337V showing global phospho-TDP staining to all the neurons and intense ubiquitin staining in the cytoplasm (DAPI negative). **D&E**. Quantification of the pTDP and Ub-K48 signal in adult brains. **D**. Quantification of phosphorylated TDP signal in the adult brains. **E**. Quantification of the Ub-K48 signal in adult brains. (ANOVA, *p<0.05,**** p<0.0001, n = 8–9). Scale bar is 50 μm in A and 2μm in B. Scale bar is 5μm.

Neurodegenerative diseases that include TDP-43 proteinopathies are usually characterized with phosphorylated TDP-43 and ubiquitin positive inclusions in the cytoplasm or the nuclei of neurons [3]. To determine whether the introduction of mutant hTDP-43 caused the phosphorylated TDP and ubiquitin-positive inclusions, adult brains were labeled with anti-phospho-TDP-43 (409/410) and antisera specific to the K48 ubiquitin linkage. Immuno-fluorescence for the ubiquitin staining of hTDP-43 WT and G294A were comparable, with no significant phospho-TDP-43 or ubiquitin puncta detected (Fig 5A & 5B). For the flies expressing hTDP-43 M337V, however, there was a global enrichment of phospho-TDP-43 signal and a higher incidence of neurons containing ubiquitin-positive puncta within the nuclei and cytoplasm (Fig 5C, 5D & 5E), similar to pathological reports in FTLD and ALS cases [3, 53]. Occasionally, we observed small ubiquitin positive puncta scattered around the nucleus, however, only rarely did they overlap with hTDP-43 puncta in the mutant lines (Figures C&D in S3 Fig).

### Only mild phenotypes are associated with expression of mutant hTDP-43

**Fig 6:**
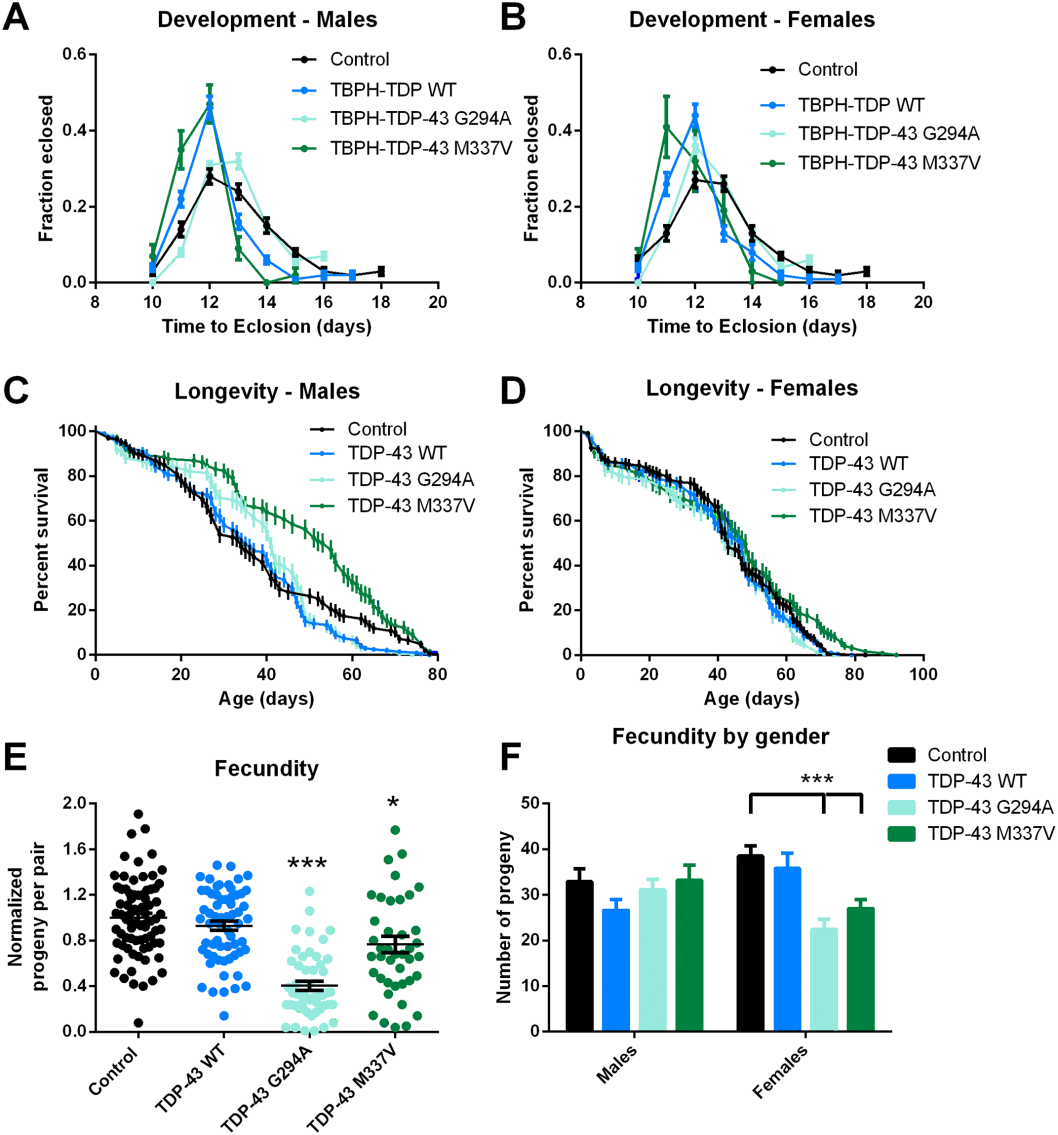
Flies that expressed mutant versions of hTDP-43 show no major phenotypes. **A&B**. Time of development. There were no significant differences in the median time taken between egg laying and eclosion between any of the lines for either male (**A**) or female (**B**) flies. Each point represents the mean ± SEM of at least 200 flies for each genotype. **C&D**. Life span. The life span of male (C) and female (D) flies were determined and showed that expression of mutant hTDP-43 did not lead to a reduced life span. For males, flies expressing hTDP-M337V had a slightly longer life span and there were no differences in the life span in female flies for any genotype. Each line represents the survival curves for between 150 and 200 flies with the error bars representing the standard error. Data was analyzed using a Mantel-Cox log rank test. **E &F**. Fecundity. Single virgin male and female flies were placed in a vial and allowed to mate and lay eggs for one week and the total number of progeny recorded. **E**. Results represent three separate experiments with males and females of the same genotype in each vial. The number of progeny from each pair was normalized to the mean number of progeny for the control flies in that experiment. Each point represents the number of progeny from a single pair of flies and the lines represent the mean and SEM for between 40 and 60 pairs of flies. Both lines expressing mutant hTDP-43 generated significantly fewer progeny than either control flies or flies expressing wild type hTDP-43, * p<0.05; *** p<0.001; ANOVA followed by Holm-Sidak multiple comparison test. **F**. Fecundity of males compared to females. Individual males of each genotype were crossed with individual females of each genotype and the total progeny of each pair were then pooled based on the sex and genotype of the parent. The data represents the mean and SEM for between 30 and 35 pairs of flies. There were no significant differences in the number of progeny between the males of each genotype, whereas female flies expressing either mutant hTDP-43 produced significantly fewer offspring. *** p<0.001; two way ANOVA followed by Sidaks multiple comparison test.

All three of the fly lines were viable as homozygotes and to determine whether there were any defects in development, we measured the length of time between hatching and adult emergence (eclosion). No significant differences were observed between control flies and those expressing wild type hTDP-43 and no delays in development were observed with flies expressing either mutant form of hTDP-43 (Fig 6A & 6B), indicating these mutations had no effect on larval or pupal development. To assess long-term survival we measured the life span of the lines and again no significant reductions were detected in any line expressing wild type or mutant hTDP-43 compared to the control lines (Fig 6C & 6D). We next set up pairwise crosses to test the fecundity of the different lines. These data showed no differences in the number of offspring produced between control flies and those expressing wild type hTDP-43, but a reduced number of progeny in the hTDP-43 G294A and M337V flies (Fig 6E). To determine whether this defect was due to defects in the male or female flies, systematic pairwise crosses between each of the lines were performed. Interestingly, no significant defect were found associated with male flies among all the lines, but a significant reduction in the number of progeny numbers produced from female hTDP-43 G294A and M337V fly lines was detected, suggesting females were more affected by the hTDP-43 mutations (Fig 6F).

**Fig 7:**
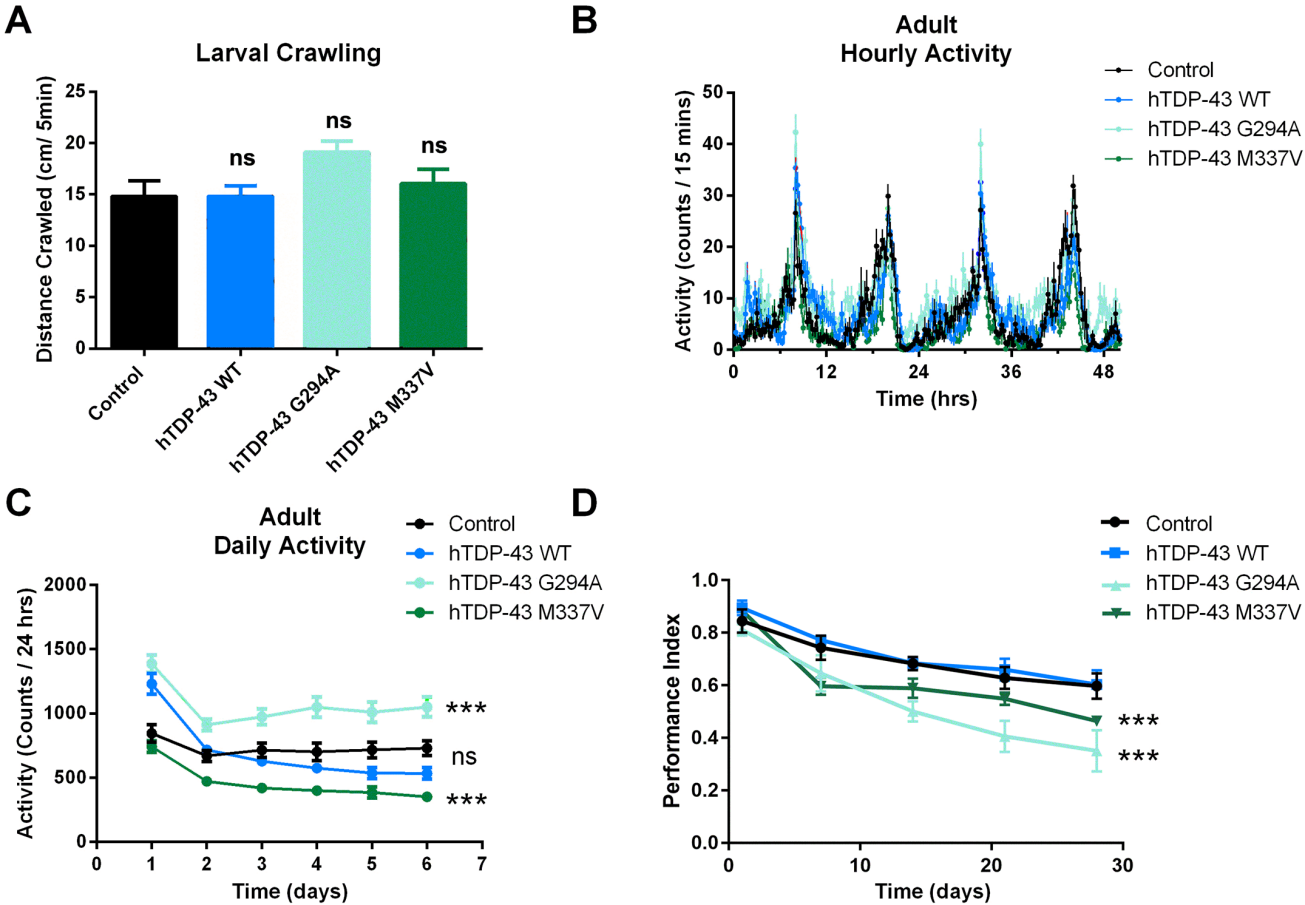
The hTDP-43 mutant flies show minor behavioral defects. **A**. Larval locomotion. The total distance third instar larvae crawled for 5 minutes on agar plate was quantified. There were no significant difference among hTDP-43 WT, hTDP-43 G294A, hTDP-43 M337V and control larvae (ANOVA, n = 21–30). **B**. Adult hourly activity. The activity pattern of individual flies was tracked using an activity recorder and no significant differences were observed for any genotype (mean ± SEM, n = 18–25 flies). **C**. Adult daily activity. The total activity for each fly was summed for each 24hr period and monitored for 6 days. There was no significant difference between control flies and flies expressing wild type hTDP-43, whereas flies expressing hTDP-43 G294A showed significantly higher levels of activity and flies expressing hTDP-43 M337V had significantly lower levels of activity compared to flies expressing wild type hTDP-43. Two way ANOVA followed by Dunnett’s multiple comparison test. Each point represents mean ± SEM n = 25–35 flies. **D**. Adult negative geotaxis. The ability of flies to climb to the top of a tube was determined as described in the methods section. There was no significant difference in the performance index of control flies and flies expressing wild type hTDP-43. The climbing indexes of hTDP-43 G294A and M337V flies were gradually reduced by time compared to that of flies expressing wild type hTDP-43. Two way ANOVA followed by Dunnett’s multiple comparison test. Each point represents mean ± SEM n = 3–20.

To determine if there were any behavioral effects of mutant hTDP-43, we analyzed larval crawling behavior by measuring the distance 3^rd^ instar larva crawled during a 5 minute observation period. No differences were detected between any of the lines (Fig 7A). To assess adult locomotion, we placed individual flies in an activity recorder that continuously monitored movement. Flies show diurnal patterns of activity and these patterns were similar in all four lines (Fig 7B). The total level of daily activity was also quantified with this approach and these results showed similar levels of activity between control flies and flies expressing wild type hTDP-43 (Fig 7C). The two mutant lines exhibited significantly different levels of daily activity with flies expressing hTDP-43 G294A showing elevated levels of activity and flies expressing hTDP-43 M337V reduced levels of activity compared to wild type hTDP-43 expressing flies (Fig 7C). Adult flies exhibit negative geotaxis behavior, rapidly climbing to the top of vials. When we measured this behavior in 1 day old flies, we detected no differences between control flies and flies expressing wild type hTDP-43, G294A and M337V. There was a gradual reduction in performance in this assay as the flies aged, with a faster decline in the fly lines expressing each of the mutant forms of hTDP-43(Fig 7D). By 4 weeks of age, both of the flies with hTDP-43 G294A and -M337V showed a significantly reduced performance, compared to the control flies and those expressing wild type hTDP-43.

## Discussion

TDP-43 cytoplasmic aggregates are a pathological characteristic of ALS, FTLD and other neurodegenerative diseases [2, 3, 5, 54]. Notably, around 90% of ALS and 50% of FTLD cases have TDP-43 aggregates, strongly implicating the formation of TDP-43 inclusions in the etiology of these diseases [55]. To mimic this TDP-43 proteinopathy, either wild type hTDP-43 or the mutant versions of this protein have been expressed in rodent models. Neurological findings such as axonal degeneration, motor neuron loss, motor dysfunction, learning/memory deficit, brain atrophy as well as TDP-43 inclusions have been reported in these studies [56–69]. Most of these models have shown that many of the phenotypes of these transgenic animals were dependent on the levels of TDP-43. Low levels of expression resulted in TDP-43 cytoplasmic accumulation and age-associated motor dysfunction [64, 70], while high levels of expression (usually 3~5 fold compared to endogenous TDP-43) caused early disease onset, but without significant motor neuron losses and TDP-43 inclusions [20]. However, there are some clinical observations concerning TDP-43 proteinopathies that have not been addressed in these models. For example, in contrast to the fast-developing disease onsets of models overexpressing TDP-43, the disease onsets of the patients with familial TDP-43 mutations range from 31 to over 70 years-old of age [26, 29, 30, 71]. Even with specific a TDP-43 mutation, e.g. M337V across three families, the average disease onsets vary from 38 to 52 years-old of age and the disability of the patients within the same pedigree varies considerably [27, 32, 33]. Similarly, diverse clinical observations are also available in other familial TDP-43 mutations [26, 28, 30, 72]. These clinical observations, however, not only indicate that the TDP-43 proteinopathies vary but also suggests that although TDP-43 mutations clearly contribute to the disease, other genetic and/or environmental factors likely play a significant role in disease onset and progression.

In the current study, we have developed a *Drosophila* system that expresses wild type and mutant hTDP-43, in the absence of endogenous TBPH, to clarify the role of mutant hTDP-43 in disease phenotypes. Although we did observe a transcriptional change in young flies (1 week-old) expressing mutant hTDP-43, however, there was no difference in transcript levels among all the older flies (4 week-old), suggesting that the mutant TDP-43 does not induce the overexpression of the gene itself. We also detected an increase in the levels of soluble hTDP-43 protein in young flies expressing mutant hTDP-43, but these differences were diminished in aged flies, as did the levels of wild-type hTDP-43. It is interesting that in young flies we detected reciprocal changes in transcript and protein levels of hTDP-43. The levels of TDP-43 protein are known to be autoregulated by binding of the TDP-43 protein to the 3’UTR of the TDP-43 transcript [73]. We hypothesize that hTDP-43 is acting through the endogenous *tbph* UTR to maintain the transcript-protein homeostasis seen in cell- and mammal models. If the mutant protein has a slower turnover rate compared to wild type protein it would result in slightly decreased transcripts in the mutant fly lines as we have observed. Our observation that hTDP-43 protein levels decrease with age is consistent with previous findings reported from both *Drosophila* and rodent models [74, 75]. Interestingly, we also observe an age-dependent reduction in mutant hTDP-43 protein levels. This reduction can be interpreted as an absence of hTDP-43 protein accumulation in these mutants. These results suggest the possibility that the accumulation of TDP-43 in the presence of familial hTDP-43 mutants [27, 76], might not simply be caused by the mutation itself, but by the involvement of other components, e.g. dysfunction of ubiquitin proteasome system [77]. In addition, interestingly, clinical results from sporadic ALS patients show that TDP-43 is not overexpressed in either its transcript or protein levels [20, 64, 78, 79].

In contrast to the relatively small differences in the proteins or transcripts among all the fly lines, the proteins immunoprecipitated with TDP-43 serum showed more substantial changes. Although the input levels of TDP-43 were equivalent across the three lines, the levels of TDP-43 immunoprecipitated were highly enriched in samples from the two mutant lines compared to the wild type hTDP-43 line, as assessed by FLAG immunoblots. This result suggests that the epitope of the wild type hTDP-43 is masked, possibly by its partner proteins, since TDP-43 usually functions as a protein complex [80]. This would suggest that these complexes are disrupted in the presence of mutant hTDP-43 allowing increased access by the antibody. In addition, in the two mutant samples, there is also an enrichment of ubiquitinated and phosphorylated hTDP-43, which parallels clinical findings [2, 3]. The increased levels of ubiquitinated proteins and phospho-hTDP-43 in neurons from flies expressing hTDP-43 M337V were also apparent from immunostaining of the CNS. Interestingly, although most, if not all neurons showed elevated levels of phospho-TDP-43, only a small fraction (less than 10%) of neurons exhibited the ubiquitin inclusions and further studies are required to determine whether these are a defined subset of neurons or whether they are random.

Unexpectedly, there were only mild behavioral defects associated with expression of mutant hTDP-43 in our lines. The lack of any differences between the flies expressing wild type hTDP-43 and control flies expressing TBPH suggests that hTDP-43 can fully fulfill all of the essential functions of TBPH. However, we expected to observe defects in flies expressing mutant hTDP-43. Development and longevity were not diminished and only minor defects in fecundity and negative geotaxis were observed. We did observe slightly increased longevity in the males expressing hTDP-43 M337V, although we do not know whether this is due to the presence of the mutant protein or as a result of differences in the genetic background of the lines, despite outcrossing all the transgenic lines with the control line. These results, nevertheless, stand in marked contrast to results from over-expression of mutant hTDP-43 in a variety of systems where it has been shown to be highly toxic [40, 41, 44, 45, 57, 64, 65, 67–70, 81–85]. This suggests that mutant forms of hTDP-43 are not inherently toxic but require a specific set of conditions that could include time, environmental or genetic interactions to manifest their deleterious effects.

## Conclusions

In summary, we have described the preliminary characterization of fly lines designed to allow the examination of the effects of TDP-43 mutations in the absence of endogenous TBPH. Flies that express mutant forms of hTDP-43 display only mild behavioral and developmental phenotypes yet show a number of cellular and molecular characteristics that appear to parallel the histopathological hallmarks of human neurodegenerative disease. These flies will be invaluable tools to identify interacting genes and the downstream components resulting from TDP-43 dysfunction. Interestingly, a recent report describes using similar CRSIPR/Cas9 technology to introduce point mutations into the TDP-43 orthologue in zebra fish, but at this time no phenotypes were reported [86].

## Supporting information

S1 Fig No age-dependent accumulation of TDP-43 in either flies expressing hTDP-43 wild type or its mutants in SDS fraction. Adult fly heads from each genotype were homogenized in Tris buffer (see Methods), centrifuged, and the supernatant (Tris fraction) analyzed by immunoblot. The pellet was re-homogenized in SDS buffer, centrifuged, and the supernatant (SDS fraction) analyzed by immunoblot in parallel. Notably, all the fly lines show an age-dependent decline in hTDP-43 levels.
(TIF)

S2 Fig The cytoplasmic puncta in neurons from flies expressing hTDP-43 M337V are not co-localized with stress granules marker, FMR1. Adult brains were fixed and stained with an anti-FLAG (green) and anti-FMR1(magenta) antiserum to label FLAG-hTDP-43 (A, B) and FMR1 (A’, B’). **A**. Wild type hTDP-43. FMR1 positive cytoplasmic puncta are clearly visible (A’). **B**. hTDP-43 M337V. Both hTDP-43 positive puncta (B) and FMR1 positive cytoplasmic puncta are clearly visible (B’) but no overlap of the two types of puncta were observed (B”). Arrows indicate the hTDP-43 containing puncta in flies expressing hTDP-43 M337V (B) and arrowheads indicate the FMR1-containing puncta. Scale bar is 2μm.
(TIF)

S3 Fig Increased ubiquitin-positive puncta in neurons in flies expressing mutant hTDP-43. Adult brains of flies expressing wild type (A) and mutant hTDP-43 (B&C) were fixed and stained with an anti-ubiquitin (gray) antiserum to label ubiquitin (A’, B’ and C’) and DAPI to label nuclei (A, B and C). Merged images are shown in the third column. **A**. Only low levels of ubiquitin staining was visible in flies expressing wild type hTDP-43. **B&C**. Flies expressing mutant hTDP-43 showed sporadic ubiquitin nuclear inclusions. Representative images from flies expressing hTDP-43 G294A (**B**) and hTDP-43 M337V (**C**) showing ubiquitin positive puncta overlapping with DAPI staining (arrows). In flies expressing hTDP-43 M337V, ubiquitin-positive puncta were also present in region not labeled with DAPI (boxed area in C”). These preparations were also stained with anti-FLAG antiserum (arrow heads in D). Examination of the merged images (D”) for both ubiquitin (arrows in D’) showed little overlap of TDP-43 and ubiquitin in these preparations. Scale bar is 2μm.
(TIF)
